# Impact on quality of life 3 years after diagnosis of prostate cancer patients below 75 at diagnosis: an observational case-control study

**DOI:** 10.1186/s12885-020-07244-y

**Published:** 2020-08-12

**Authors:** Nadine Houédé, Xavier Rébillard, Sophie Bouvet, Sarah Kabani, Pascale Fabbro-Peray, Brigitte Trétarre, Florence Ménégaux

**Affiliations:** 1 Institut de Cancérologie du Gard, CHU Nîmes, Rue du Pr Henri Pujol, 30029 Nîmes Cedex 9, France; 2 INSERM U1194, Montpellier Cancer Research Institute & Université de Montpellier, Montpellier, France; 3 Urology Department, Clinique Beau Soleil, Montpellier, France; 4 Department of Biostatistics, Epidemiology, Public Health and Innovation in Methodology, (BESPIM), CHU Nîmes, Nîmes, France; 5 Registre des Tumeurs de l’Hérault, ICM, Montpellier, France; 6 Center for Research in Epidemiology and Population Health, INSERM U1018, Villejuif, France

**Keywords:** Prostate cancer, Quality of life, Long term survival, Side effects, Urinary dysfunction

## Abstract

**Background:**

Prostate cancer patients are known to suffer from poor sexual and urinary long-term side-effects following treatment, potentially impacting quality of life. The purpose of our study was to compare health-related quality of life at 3 years between prostate cancer patients and healthy controls according to key life-style characteristics. Secondary objectives were to compare urological dysfunction, sexual function, anxiety and depression.

**Methods:**

Multicentric, case-control, observational prospective, open, follow-up study including 819 prostate cancer patients < 75 years old from the EPICAP cohort, newly diagnosed from 1 December 2011 to 31 March 2014 and 879 healthy controls. Participants were excluded if they experienced a relapse. Controls from the same geographical region were age-matched and were excluded if they were diagnosed with prostate cancer. Patients received one of the following treatments: active surveillance (AS), radical prostatectomy (RP), external beam radiotherapy (EBRT), High-intensity Focused Ultrasound (HIFU), chemotherapy (CT), or androgen deprivation therapy (ADT) as appropriate. The primary outcome was the quality of life as evaluated by the QLQ-C30 questionnaire. Scores were analyzed by multivariate analysis to adjust for predefined socio-demographic confounding effects.

**Results:**

In total, 564 participants were included (mean age 67.9 years): 376 patients and 188 controls. Treatment breakdown was: 258 underwent RP, 90 received EBRT, 52 brachytherapy or HIFU, 15 CT, 26 ADT and 61 AS. There was no difference in median global quality of life between patients and controls (94.87 vs 94.15, *p* = 0.71). Multivariate analysis showed poorer social functioning in patients (24.3% vs. 16.3%, *p* = 0.0209), more dyspnea (22% vs. 12.4%, *p* = 0.0078), and yet less current pain (23% vs 33%, *p* = 0.0151).

**Conclusions:**

Global health status score at 3 years after diagnosis was similar between patients and controls, though patients showed a significantly worse social functioning. Prostate cancer diagnosis per se does not seem to impact the quality of life of patients < 75 years at diagnosis. However, the therapeutic option that will be chosen following diagnosis should be carefully discussed with the medical staff in terms of benefit-risk ratios as it could have a long-term impact on urinary or erectile dysfunction.

**Trial registration:**

clinicaltrials.gov, NCT02854982. Registered 4 August 2016, retrospectively registered.

## Background

Prostate cancer (PCa) diagnosis and treatment are known to impact patient short-term quality of life (QoL) and functional symptoms [1]. However, longer-term effects also need to be considered to choose the most adapted treatment and anticipate rehabilitation difficulties. Studies examining QoL following treatment have shown worsening of sexual and urinary troubles over time [2–4]. A large study showed that half of patients included 2 years after diagnosis experienced negative consequences of PCa and treatment, with a negative impact on QoL [5]. A French study found that 20% of treated patients had erectile dysfunction and over 10% were treated for acute urinary retention; both dysfunctions occurred at higher frequency in patients treated with prostatectomy alone [6].

A recent review showed that QoL of newly diagnosed PCa patients was independent of the type of treatment, but that surgery had a negative impact on urinary continence and sexual function, and external beam radiotherapy (EBRT) on bowel function; active surveillance (AS) had the lowest impact on disease-specific QoL [7]. These studies focused on treatment-related clinical symptoms, but did not consider the natural aging process despite age-related comorbidities possibly interacting with the adverse effects of different treatment modalities.

Our study investigated QoL following PCa in the EPICAP cohort [8] to evaluate its potential deterioration and the occurrence of long-term sexual or urinary dysfunctions that could arise from natural aging of the population.

## Methods

Study design and setting: EPICAP-QALY is an ancillary study of the EPICAP survey [8]. EPICAP is a multicentric case-control, observational prospective, open, follow-up study including newly diagnosed PCa patients between 2012 and 2014 (819 patients) and 879 age-matched healthy controls from the same area. The EPICAP-QALY was performed at Nimes University Hospital between August 2015 and October 2017 and approved by the institutional review board.

Participants: All participants from the EPICAP cohort completed a screening questionnaire to determine eligibility. Patients who had received hormone therapy within the previous year or who experienced a relapse in the intervening years were excluded, except patients on salvage radiotherapy following prostatectomy for more than 6 months with a PSA level < 1 ng/ml. Age-matched ±1 year healthy controls were included in a 1:2 ratio. Individuals diagnosed with PCa following inclusion or with a PSA > 10 ng/ml were excluded. Men with PSA > 10 ng/ml at the time of completing the questionnaire were not selected to exclude potential relapse for cases or cancer occurrence for controls.

Outcomes: The primary outcome was QoL 3 years after PCa treatment compared to controls, as evaluated by the QLQ-C30 questionnaire [9]. Secondary outcomes were the comparison of urinary, sexual and anxiodepressive dysfunction between patients and controls using the following questionnaires: IPSS International Prostate Symptom Score [10], ICIQ-MLUTS International Consultation on Incontinence Male Lower Urinary Tract Symptoms [11], IIEF-6 International Index of Erectile Function [12], and HADS Hospital Anxiety and Depression Scale [13].

These questionnaires were used to compare QoL and symptoms according to active surveillance (AS), radical prostatectomy (RP), EBRT, brachytherapy or High-intensity Focused Ultrasound (HIFU), androgen deprivation therapy (ADT) or combined care (CC). A life situation questionnaire complemented with specific questions concerning sexuality was used to test for some potential confounders [14].

Data collection: Age, BMI, PSA level, educational level, housing, living alone, marital status, monthly income, chronic disease and regular medication were collected. Treatment at diagnosis, last treatment received, hormone therapy within previous 12 months, and employment status were also recorded. For controls, urologic consultation for urinary troubles, prostate treatment and PSA testing in the 3 previous year were recorded.

Sample size: By predicting a lower participation rate in cases than controls and a recurrence rate of cases of 10%, we originally planned a cohort of 600 patients and 300 controls paired with a ratio 2:1 on age to highlight a standardized difference in score on the QLQ-C30 of 0.25 (“small” effect according to Cocks et al. [9]) with a global bilateral risk alpha of 5 and 90% power. The participation rate was lower than expected and the study included 376 patients to whom we matched 188 patients (from the 364 available).

Statistics: The comparability of age was assessed with a Student test. Descriptive statistics are reported as counts and percentages for categorical variables and means and standard deviations for continuous variables with normal distribution and median and quartiles for others. Comparisons of baseline characteristics and putative risk factors between cases and controls were performed with Mann–Whitney, Kruskal–Wallis, χ2, Student, or Fisher exact test as appropriate.

For each questionnaire, the distribution of scores was analyzed. When extreme values (0 or 100) were over-represented, scores were recoded into classes and described qualitatively with effectives and percentages.

The univariate analysis was performed with a mixed linear model for quantitative scores (QLQ-C30 summary score, VS and IS score of ICIQ-MLUTS). For recoded QLQ-C30 scores, analyses were conducted with a mixed logit model. To account for pairing, a random effect on 2: 1 trinoma was considered.

For recoded QLQ-C30 scores, distribution and links with social potential confounders was assessed. When the symptom or trouble was present in less than 20% of cases or when no apparent link was possible, multivariate analysis was not performed. For other QLQ-C30 scores and for the summary quantitative score, the effects of putative confounders were evaluated. Socio-professional integration items were selected for testing based on their reliability, their clinical pertinence of potential confounding factors and their similarity with items of the QALIPRO study [15]. Putative confounders for quantitative scores were analyzed with Spearman correlation test, Kruskal-Wallis or ANOVA as appropriate, and with χ2, Fisher test, Student or Wilcoxon test for qualitative values.

All variables with a *P*-value lower than 0.20 were considered as potential covariates and adjusted mixed linear general models or logistic models were computed with a random effect on 2:1 trinoma.

All analyses were performed using SAS software (SAS Institute, Cary, NC) version 9.3. *P*-values < 0.05 were interpreted as statistically significant for 2-sided tests. Since multiple comparisons increase the risk of introducing a Type-I error, we applied the sequentially rejective Bonferroni correction (Holm’s correction) to control for this type of error in Tables 2 and 3. This means that the *p*-value must be divided by the number of tests run in parallel, resulting in an adjusted level of statistical significance. The corrected *p*-values for Holm’s correction are reported. For multivariate analysis, Holm’s correction is also applied on p-values of interest obtained by the models.

## Results

**Fig. 1:**
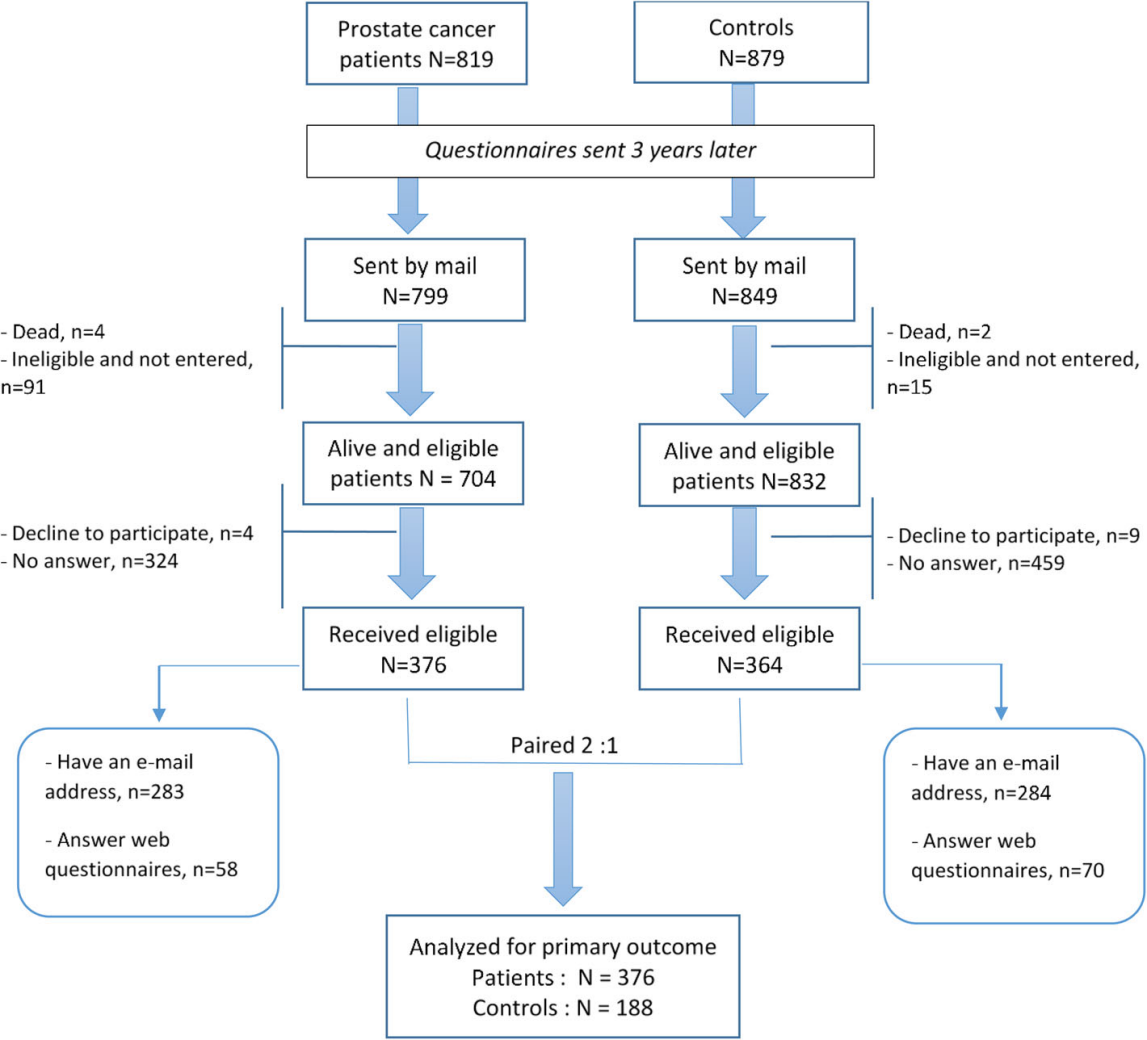
Flow chart

Between August 2015 and October 2017, questionnaires were sent to the 799 patients and 849 controls from the EPICAP cohort for whom a postal address was available. Among these 1648 subjects, 6 had died and 106 were non-eligible for the EPICAP-QALY study. Responses to questionnaires were received from 376/704 eligible patients (53.4%) and 364/832 eligible controls (43.8%) (Fig. 1). Patient profiles did not significantly differ between participants and non-participants to the study according to age, Gleason score or BMI (Supplementary Table). The controls were age-matched in a 2:1 ratio with the patients (*n* = 188). The average patient age was 67.9 years old. Baseline characteristics at inclusion were similar between groups, except for PSA level, which was much lower in the patient group as anticipated due to treatment (Table 1). The most common treatments for patients were radical prostatectomy (RP) (68.6%) and EBRT (23.9%).

**Table 1:** Patient and control baseline characteristics

Population description			*p*-value
	Patients *N* = 376	Controls *N* = 188	
**Age (years)**	67.9 ± 5.8	67.9 ± 5.8	
**BMI (kg/m**^**2**^**)** **Missing data**	26.7 ± 3.4 6	26.6 ± 3.8 3	0.70
**PSA levels (ng/ml)** **Missing Data**	0.03 [0.01–0.2] 15	1.52 [0.75–3.11] 55	
**Educational level**			
**Secondary school**	161 (43.4%)	84 (45.7%)	0.26
**University**	121 (32.6%)	67 (36.4%)	
**Post-graduate**	89 (24%)	33 (17.9%)	
**Missing**	5	4	
**Living alone**	28 (7.6%)	19 (10.3%)	0.27
**Marital status**			0.29
**Single**	15 (4%)	6 (3.2%)	
**Married / in couple**	333 (89%)	161 (86.6%)	
**Separated**	16 (4.3%)	8 (4.3%)	
**Widowed**	10 (2.7%)	11 (5.9%)	
**Missing data**	2	2	
**Monthly income**			
**0 to 750 €**	12 (3.4%)	4 (2.2%)	0.81
**750 to 1500 €**	30 (8.5%)	17 (9.4%)	
**1500 to 3000 €**	149 (42%)	80 (44.4%)	
**> 3000 €**	164 (46.2%)	79 (43.9%)	
**Missing data**	21	8	
**Chronic disease**			0.33
**None**	150 (42.6%)	66 (38.2%)	
**At least one**	202 (57.4%)	107 (61.9%)	
**Missing data**	24	15	
**Regular medication** **Missing data**	256 (70%) 10	134 (72.8%) 4	0.48
Treatment strategy at diagnosis*			
**Active surveillance**	61 (16.2%)	–	
**Radical prostatectomy**	258 (68.6%)	–	
**Chemotherapy**	15 (4%)	–	
**EBRT**	90 (23.9%)	–	
**Brachytherapy or HIFU**	52 (13.8%)	–	
**Androgen deprivation therapy**	26 (6.9%)	–	
**Missing data**	5	–	
**Patient in active employment at diagnosis** **Missing data**	71 (19%) 3	–	
**Patient in active employment at time of questionnaire** **Missing data**	50 (13.5%) 5	–	

Data are given as average ± standard deviation, median [IQR] or number (%) as appropriate. *HIFU* High Intensity Focused Ultrasound, *EBRT* external beam radiotherapy

*Certain patients received combined treatments

**Fig. 2:**
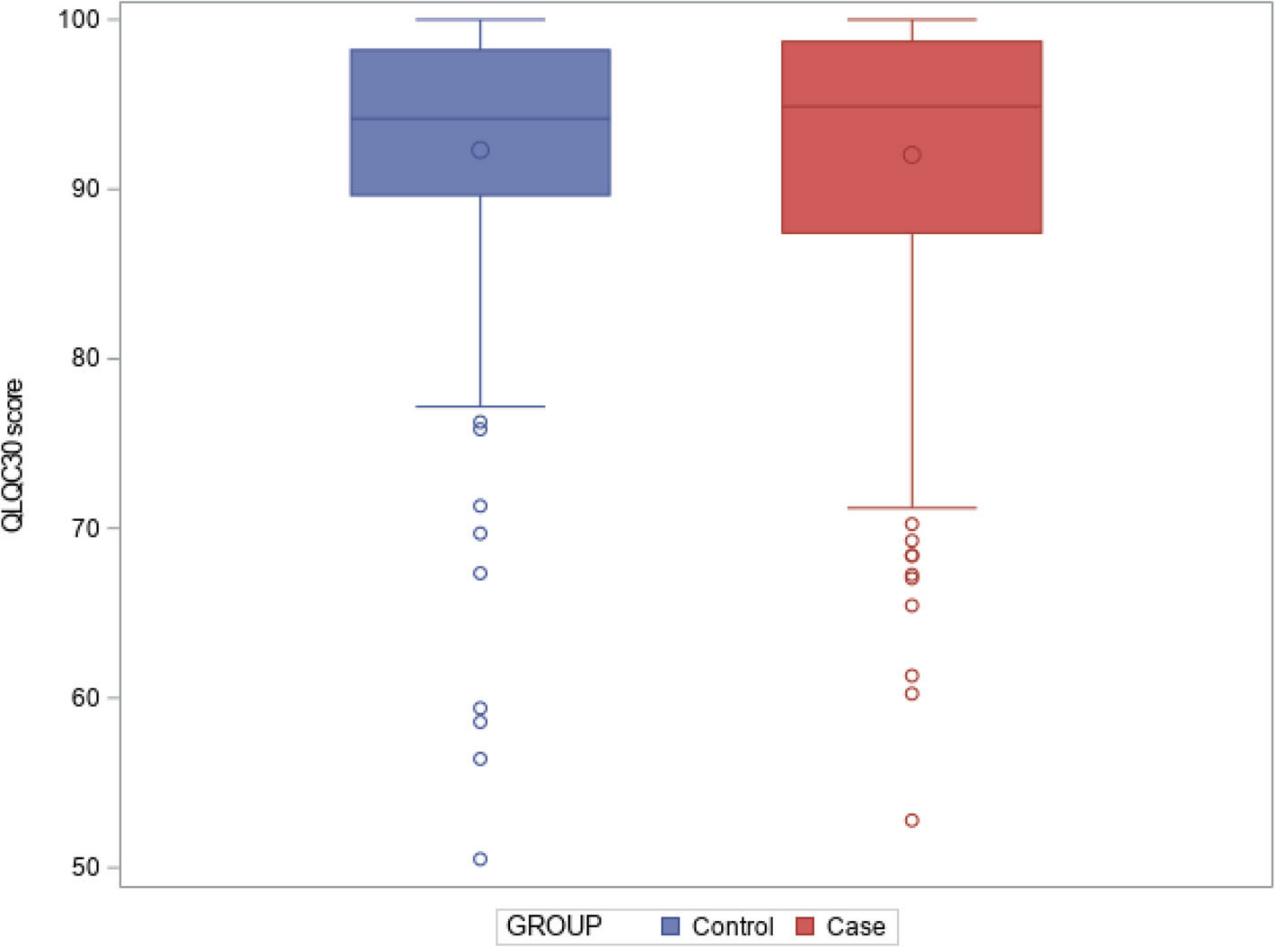
Boxplot of QLQ-C30 summary score between patients and controls

Primary outcome: QLQ-C30 scores were high and did not differ between groups (Fig. 2); median summary scores were respectively 94.87 [87.44; 98.72] and 94.15 [89.66; 98.21] for patients and controls, *p* = 1 (Table 2). No significant difference in the QLQC30 was highlighted in univariate analysis.

**Table 2:** Comparisons of quality of life and symptoms between patients and controls: Results from questionnaires

Score	Patients ***N*** = 376	Controls ***N*** = 188	***p***-value
QLQ-C30			
QLQ-C30 Global score, median [Q1;Q3]	94.87 [87.44; 98.72] 11 DM	94.15 [89.66; 98.21] 4 DM	0.71
Global health status/QoL: Worse global health (<=83.3), n(%)	117 (31.3) *2 DM*	49 (26.5) *3 DM*	0.25
Physical Functioning: score < 100, n (%)	136 (36.5) *3 DM*	63 (34.1) *3 DM*	0.58
Role Functioning: score < 100, n (%)	71 (19) *3 DM*	25 (13.5) *3 DM*	0.11
Emotional Functioning: score < 100, n(%)	204 (54.6) *2 DM*	89 (48.1) *3 DM*	0.15
Cognitive Functioning: score < 100, n(%)	170 (45.6) *3 DM*	99 (53.5) *3 DM*	**0.0799†**
Social Functioning: score < 100, n (%)	91 (24.3) *2 DM*	30 (16.3) *4 DM*	0.0332*
Fatigue: score > 0, n (%)	167 (44.7) *2 DM*	84 (45.4) *3 DM*	0.87
Nausea/Vomiting: score > 0, n (%)	16 (4.3) *2 DM*	6 (3.2) *3 DM*	0.56
Pain: score > 0, n (%)	86 (23) *2 DM*	61 (33) *3 DM*	0.0131*
Dyspnea: score > 0, n(%)	82 (22) *4 DM*	23 (12.4) *3 DM*	0.0078*
Insomnia: score > 0, n (%)	128 (34.2) *2 DM*	70 (37.8) *3 DM*	0.40
Appetite loss: score > 0, n (%)	19 (5.1) *4 DM*	11 (6) *3 DM*	0.68
Constipation: score > 0, n (%)	69 (18.5) *3 DM*	44 (23.8) *3 DM*	0.15
Diarrhea: score > 0, n (%)	53 (14.3) *5 DM*	24 (13) *3 DM*	0.67
Financial Problems: score > 0, n (%)	22 (6) *10 DM*	6 (3.3) *5 DM*	0.18
IPSS			
Mild	254 (72.4)	141 (78.8)	0.107
Moderate	77 (21.9)	31 (17.3)	
Severe	20 (5.7) 25 DM	7 (3.9) 9 DM	
IIEF-6			
Severe erectile dysfunction	209 (65.5)	55 (32)	< 0.0001
Moderate dysfunction	28 (8.8)	19 (11.2)	
Mild to moderate dysfunction	26 (8.2)	13 (7.6)	
Mild dysfunction	20 (6.3)	26 (15.1)	
No dysfunction	36 (11.3) 57 DM	59 (34.3) 16 DM	
ICIQ-MLUTS			
Voiding score (VS)	2 [0; 5] 17 DM	2 [1; 5] 6 DM	0.78
Incontinence score (IS)	3 [1; 6] 16 DM	2 [0; 3] 5 DM	**< 0.0001**
Frequency of diurnal urination			0.0884
- 1–6 times per day	213 (58.4)	124 (66.3)	
- 7–8 times per day	107 (29.3)	43 (23)	
- ≥9 times per day	45 (12.3) 11 DM	20 (10.7) 1 DM	
Frequency of nocturnal urination			0.99
- Never	74 (20.1)	33 (17.9)	
- 1 time per night	191 (51.9)	93 (50.5)	
- ≥ 2 times per night	103 (28) 8 DM	58 (31.5) 4DM	
HADS Anxiety			
Absence of anxiety	266 (74.9)	140 (77.4)	0.64
Suspected anxiety	59 (16.6)	31 (17.1)	
Probable anxiety	30 (8.5) 21 DM	10 (5.5) 7 DM	
HADS Depression			
Absence of depression	303 (85.1)	155 (87.6)	0.44
Suspected depression	36 (10.1)	15 (8.5)	
Probable depression	17 (4.8) 20 DM	7 (4) 11 DM	
HADS Total score			
Absence of anxio-depressive troubles	277 (80.8)	149 (85.6)	0.17
Presence of anxio-depressive troubles	66 (19.2) 33 DM	25 (14.4) 14 DM	

Data presented as number (%) or median [IQR] as appropriate. Results presented as number (%) patients in each group with functional scores < 100 and symptom scores as > 0. DM: Data missing; *significant difference (< 5%); †potentially significant difference (5–10%)

Estimation of confounding factors in the multivariate regression model could only be performed for QLQ-C30 summary score, global health status/QOL, emotional functioning, cognitive functioning, social functioning, fatigue, pain and insomnia, for which enough data were collected. Multivariate analysis of QLQ-C30 summary score was conducted on 540 participants using the following variables: age, group (patient vs. control), type of lodging, and presence of a chronic illness. No significant difference of QLQ-C30 summary score was observed between groups (*p* = 1). For global health status/QOL, emotional functioning, cognitive functioning, fatigue and insomnia, multivariate analysis confirmed the absence of difference shown in univariate analysis with a reduced level of statistical significance using Holm’s correction, with respectively *p* = 1, *p* = 1, *p* = 0.91, *p* = 1, *p* = 1, *p* = 0.29, *p* = 0.23 for global health status/QOL, emotional functioning, cognitive functioning, fatigue and insomnia, social functioning and pain.

Secondary outcomes: The univariate analysis showed no difference between medical care for QLQ-C30 scores between treatments.

Responses to the IPSS questionnaire showed no significant difference between patients and controls for urinary symptoms: the median score was 4 [2–8] for patients vs 3 [1–7] for controls, with the majority of subjects in each group classified as suffering from mild urinary symptoms (72.4% patients vs. 78.8% controls; *p* = 1) (Table 2). The results remain non-significant when adjusted for age (*p* = 1).

The ICIQ-MLUTS questionnaire showed no difference in voiding score (VS) between patients and controls (median of 2 [0; 5] and 2 [1; 5] respectively; *p* = 1), but a significantly higher incontinence score (IS) in patients (median score 3 [1; 6] vs. 2 [0; 3] respectively, *p* = 0.0025). Age adjustment confirmed these results. Frequency of diurnal and nocturnal urination did not differ between groups (*p* = 1 for both). The majority of subjects in both groups reported frequency of urination between 1 and 6 times per day (58.4% for patients vs. 66.3% controls), with only 12.3% patients and 10.7% controls reporting a frequency ≥ 9 times per day. Similarly, for night frequency, patients and controls mostly reported 1 voiding per night (51.9% vs. 50.5% respectively). Only 4 questions about bladder weakness (questions 8, 9, 10 and 12) showed differences between patients and controls in terms of level of bother experienced.

In contrast, the IIEF-6 questionnaire revealed a significant difference (*p* = 0.0025) in erectile dysfunction between groups with 65.5% of patients having severe erectile dysfunction compared to 32% of controls. Only 11.3% patients were free of dysfunction versus 34.3% of controls. Adjustment for age did not affect the significance of the results.

The HADS questionnaire showed no difference in the number of participants with probable anxiety and/or depression between groups. Median HADS total score was 8 [5; 13] for patients versus 9 [6; 13] for controls, thus 80.8% patients and 85.6% controls showed absence of anxio-depressive symptoms (*p* = 1).

QLQC30, IPSS, IIEF-6, HADS and ICIQ-MLUTS scores were analyzed in 370 out of 376 patients (medical care of 5 patients was missing, and 1 patient treated with hormonotherapy alone was not eligible) according to the different treatments: 10.5% (*n* = 39) in AS, 50.5% (*n* = 187) with a RP alone, 14.6% (*n* = 54) with EBRT, brachytherapy, HIFU or ADT and 24.3% (*n* = 90) with CC.

No significant difference was highlighted for QLQC30 scores (Table 3). The IPSS score, frequency of day or night urination and HADS scores did not differ between the different medical cares. For the ICIQ-MLUTS questionnaire, VS score and IS score were both significantly different (*p* = 0.00253 and *p* = 0.0025, respectively) between treatments and no potential confounder was highlighted. The Bonferonni-post-hoc-analysis (threshold *p* <  0.0083) showed significant differences between RP and EBRT or brachytherapy or HIFU or ADT for VS score (*p* = 0.0009) and IS score (*p* ≤ 0.0001), whereby VS and IS scores were lowest for patients treated with RP and RT, respectively. IS score was significantly different between AS and RP (*p* = 0.0013).

**Table 3:** Comparisons of quality of life and symptoms between the different medical care of patients: Results from questionnaires

Score	Active surveillance	Radical prostatectomy	Radiotherapy or Brachytherapy or HIFU or ADT	Combined care	*p*-value
QLQ-C30					
Summary score QLQ-C30	97.44 [94.83; 100]	95.51 [87.65; 98.72]	92.31 [87.44; 97.78]	93.25 [84.44; 98.72]	**0.0192**
Global health status/QOL: Poorer global health (<=83.3), n (%)	*6 (15.4)*	58 (31.2)	18 (34)	35 (38.9)	0.0694
Physical Functioning: score < 100, n (%)	10 (25.6)	60 (32.3)	27 (50.9)	36 (40.5)	0.0319
Role Functioning: score < 100, n (%)	5 (12.8)	37 (19.9)	11 (20.8)	17 (19.1)	0.76
Emotional Functioning: score < 100, n (%)	18 (46.2)	102 (54.8)	28 (52.8)	53 (58.9)	0.60
Cognitive Functioning: score < 100, n (%)	11 (28.2)	79 (42.7)	27 (50.9)	51 (56.7)	0.0153
Social Functioning: score < 100, n (%)	5 (12.8)	46 (24.7)	12 (22.6)	28 (31.1)	0.1671
Fatigue: score > 0, n (%)	12 (30.8)	88 (47.3)	28 (52.8)	38 (42.2)	0.1589
Nausea/Vomiting: score > 0, n (%)	1 (2.6)	5 (2.7)	2 (3.8)	8 (8.9)	0.1320*
Pain: score > 0, n (%)	9 (23.1)	42 (22.6)	12 (22.6)	23 (25.6)	0.96
Dyspnea: score > 0, n (%)	6 (15.4)	37 (20)	17 (32.1)	22 (24.7)	0.1834
Insomnia: score > 0, n (%)	10 (25.6)	60 (32.3)	20 (37.7)	36 (40)	0.36
Appetite loss: score > 0, n (%)	1 (2.6)	10 (5.4)	2 (3.9)	6 (6.7)	0.88*
Constipation: score > 0, n (%)	1 (2.6)	38 (20.5)	9 (17)	17 (18.9)	0.0640
Diarrhea: score > 0, n (%)	7 (18)	23 (12.5)	11 (21.2)	12 (13.3)	0.40
Financial Problems: score > 0, n (%)	2 (5.3)	11 (6)	2 (3.9)	7 (8.1)	0.80*
IPSS					
Mild	23 (63.9)	137 (76.5)	28 (60.9)	63 (75)	
Moderate	9 (25)	37 (20.7)	13 (28.3)	15 (17.9)	0.0730
Severe	4 (11.1)	5 (2.8)	5 (10.9)	6 (7.1)	
ICIQ-MLUTS					
Voiding score (VS)	4 [1; 7]	2 [0; 4]	3.5 [2; 6]	3 [1; 5]	0.0011
Incontinence score (IS)	3 [0; 4]	4 [2; 6]	2 [1;4]	2 [1.5; 6]	< 0.0001
Frequency of diurnal urination					
- 1–6 times per day	22 (56.4)	106 (59.2)	30 (55.6)	51 (58.6)	
- 7–8 times per day	13 (33.3)	50 (27.9)	17 (31.5)	26 (29.9)	0.99
- ≥9 times per day	4 (10.3)	23 (12.9)	7 (13)	10 (11.5)	
Frequency of nocturnal urination					
- Never	7 (18)	44 (24.3)	8 (15.1)	15 (16.9)	
- 1 time per night	19 (48.7)	89 (49.2)	23 (43.4)	55 (61.8)	0.0925
- ≥ 2 times per night	13 (33.3)	48 (26.5)	22 (41.5)	19 (21.4)	
IIEF-6					
No dysfunction	9 (23.1)	11 (5.9)	5 (9.3)	9 (10)	
Mild, Mild to moderate, or Moderate dysfunction	17 (43.6)	25 (13.4)	14 (25.9)	16 (17.8)	< 0.0001
Severe Erectile dysfunction	13 (33.3)	151 (80.8)	35 (64.8)	65 (72.2)	
HADS					
HADS anxiety					
- Absence of anxiety	31 (81.6)	124 (71.3)	42 (82.4)	64 (74.4)	
- Suspected anxiety	6 (15.8)	32 (18.4)	7 (13.7)	13 (15.1)	0.49
- Probable anxiety	1 (2.6)	18 (10.3)	2 (3.9)	9 (10.5)	
HADS depression					
- Absence of anxiety	33 (86.8)	145 (81.5)	44 (89.8)	75 (88.2)	
- Suspected anxiety	5 (13.2)	19 (10.7)	3 (6.1)	9 (10.6)	0.1588
- Probable anxiety	0	14 (7.9)	2 (4.1)	1 (1.2)	
HADS Total score					
- Absence of anxio-depressive troubles	33 (86.8)	130 (76.9)	40 (85.1)	69 (83.1)	0.34
- Presence of anxio-depressive troubles	5 (13.2)	39 (23.1)	7 (14.9)	14 (16.9)	

Data presented as number (%) or median [IQR] as appropriate. * Fisher test; Khi^2^ test otherwise

Erectile dysfunction differed between groups, with severe erectile dysfunction for 33.3, 80.8, 64.8 and 72.2% respectively for AS, RP, EBRT or brachytherapy or HIFU or ADT and CC. The analysis with the 5 classes of erectile dysfunction could not be tested, but when grouping into 3 classes (No dysfunction; Mild, Mild to moderate or Moderate dysfunction; Severe dysfunction), the Bonferroni-Holm corrected *p*-value confirmed a difference (*p* = 0.0025). Multivariate analysis was not performed due to insufficient patients with no dysfunction.

## Discussion

Three clinical trials, have shown equivalent OS between EBRT, RP and AS in low-risk prostate cancer subjects [16–18]. The ProtecT trial showed no difference in OS 10 years after diagnosis irrespective of treatment [18]. However, the impact of each of these approaches in terms of QoL and long-term side-effects remained unclear.

Our study was particularly adapted to assess the impact of natural aging, diagnosis and treatment on QoL at 3 years after diagnosis. For 376 patients and 188 age-matched controls adjusted for socio-demographic confounding variables, QoL was similar between patients and controls, with a very high QoL and few reported symptoms. Most QoL items were equivalent between groups, except worse social functioning in patients compared to controls, probably linked to side-effects. However, anxiety and depression were not different between patients and controls.

Because QoL is affected by various socio-demographic factors [19], its evaluation requires a dedicated control cohort to minimize potential biases [20]. However, only a few studies evaluating QoL and symptoms of PCa patients were performed using such a control group [16, 17, 21, 22]. Taylor et al. [23] showed a significant persistence of long-term treatment-related sexual and urinary adverse effects in PCa patients vs unmatched healthy controls. These adverse effects were observed between 5 and 10 years post-diagnosis, but global QoL was not evaluated. Kerleau et al. showed that QoL among PCa survivors 10 years post-diagnosis was equivalent to a control group from the general population [15].

Previous studies using the QLQ-C30 questionnaire for PCa reported a score of approximately 70 (out of 100) for global QoL in a Finnish and a German population [24] and 80 in a French population [15]. The unexpected high QoL and low symptoms scores observed in EPICAP-QALY could be attributed to the relatively young age and high level of income of our population but also to coping and adjustment [25]. In an extensive review, including 18 studies on PCa patients, 5 categories of strategies to adapt to illness situation were described: minimization, directing cognition and attention, reframing masculinity, retain pre-illness lifestyle and symptom management. All these attitudes can positively affect the quality of life, even a long time after therapeutic care.

There was heterogeneity in patients’ characteristics as our study was not randomized. Patients undergoing radiotherapy are usually older, have more co-morbidities and a more aggressive disease [26]. When developing individualized prediction models for the outcomes (relapse or death), some patient characteristics are associated with different treatment-related outcomes, for example reduced mortality rate in patients with elevated Gleason score choosing EBRT, whereas patients with perineural invasion fared better following surgery [26]. Nevertheless, there is a difference in long-term side-effects between the different treatments. In our study, global QoL and physical functioning scores were higher for AS and lower for ADT, with a negative impact on cognitive functions.

Incontinence, bowel dysfunction and erectile dysfunction were the main consequences of treatment. Treatment-related incontinence and erectile dysfunctions appear in the first years of treatment and persist over time with a severity that varies according to treatment [23]. In our study patients showed an increased prevalence of incontinence compared to controls, though other markers of urinary dysfunction were similar between groups, in particular following RT. Patients had worse incidence of severe erectile dysfunction compared to controls (88.5% vs 55%), especially following surgery.

In a previous study, long-term change in urinary incontinence was worse for patients treated with RP compared to brachytherapy, while long-term change in urinary irritation/obstruction was worse for patients treated with EBRT or brachytherapy [27]. Two other studies with 2 and 3 years of follow-up found that patients experienced worse sexual function and urinary incontinence after RP, worse urinary irritation/obstructive symptoms after RT, and mostly transient declines in bowel function after EBRT [28, 29]. Erectile dysfunction was observed in 87.0 and 93.9% of patients following RP and RT, respectively [30] despite a significant difference in the prevalence of urinary incontinence (18.3% vs 9.4%, respectively). In the ProtecT trial [1, 18] the surgery group reported worse urinary function. Proportion of long-term bowel dysfunction as evaluated by the EPIC questionnaire was higher in the EBRT group [1], while no difference between subgroups was observed in our study. In the CAESAR+ study [31], patients treated with RP or EBRT reported better QoL than patients receiving combined treatments, while two other studies showed that global QoL did not significantly differ in the long-term, irrespective of treatment [29, 32]. Two other studies have also looked at the long-term sequelae of the management of PCa. The first one was a Scandinavian study comparing patients of the SPCG-4 study randomly assigned to RP or watchful waiting and deferred endocrine treatment between 1989 and 1999 to a population-based control group matched for region and age [22]. Evaluation at 12 years post-diagnosis found a higher rate of erectile dysfunction (84 and 46%) and incontinence (41 and 11%) in men randomized to RP. However, a direct comparison of these data to our results is difficult as the patients were younger than in our study and were probably treated with older methods of prostatectomy, and because different questionnaires were used. The other case-control study from the Swedish register found very similar results, also using the IPSS score, with 33% incontinence for patients treated in combination and 20% for prostatectomy alone, 9% for RT alone and 73% of patients treated with erectile dysfunction including 62% post prostatectomy [21].

Our study has several limitations. It is not a prospective study, so urinary and erectile dysfunction and QoL data at baseline are missing. Since assessment was performed 3 years after diagnosis, it could be possible that QoL may have been much reduced within the first 2 years. The social-professional questionnaire was developed for testis patients usually younger and in employment, but provided necessary information for use in the statistical models as well as insights into the implication of diagnosis on working prospects [14]. Because the treatment was not randomized, some confounding factors could have an impact on side-effects between patient subgroups. It is also likely that non-responders had a different opinion on their medical care. Finally, the study design did not allow access to the patient medical files, preventing subgroup analysis according to treatment.

## Conclusion

Our study comparing health-related quality of life at 3 years after diagnosis suggests that, for PCa patients below 75 years old, the diagnosis of prostate cancer has only a marginal impact on the quality of life and on symptoms, which is an additional argument in favor of PCa screening. Conversely, our data support the hypothesis that the choice of the therapeutic option could affect social functioning due to the potential occurrence of long-term side effects.

## Supplementary information

**Additional file 1: Table S1.** Profile of subjects from the EPICAP cohort contacted and included in this study.

## Data Availability

The datasets used and/or analyzed during the current study are available from the corresponding author on reasonable request.

## Abbreviations

ADT: Androgen Deprivation Therapy
AS: Active Surveillance
BMI: Body Mass Index
CC: Combined Care
CT: Chemotherapy
EBRT: External Beam Radiotherapy
HADS: Hospital Anxiety and Depression Scale
HIFU: High-intensity Focused Ultrasound
IS: Incontinence Score
ICIQ-MLUTS: International Consultation on Incontinence Male Lower Urinary Tract Symptoms
IIEF-6: International Index of Erectile Function-6
OS: Overall Survival
PFS: Progression Free Survival
PCa: prostate cancer
PSA: Prostate Specific Antigen
RP: Radical Prostatectomy
QoL: Quality of Life
VS: Voiding Score
