# Supplementary material for: Impact on quality of life 3 years after diagnosis of prostate cancer patients below 75 at diagnosis: an observational case-control study
Source: BMC Cancer. 2020 Aug 12;20:757. doi: 10.1186/s12885-020-07244-y (PMC7424648; doi:10.1186/s12885-020-07244-y)
Supplement: Supplementary file 1 — Additional file 1: Table S1. Profile of subjects from the EPICAP cohort contacted and included in this study. [file 12885_2020_7244_MOESM1_ESM.docx]

|  | EPICAP cohort | | Contacted for inclusion | | Included in study | |
| --- | --- | --- | --- | --- | --- | --- |
|  | Patient | Control | Patient | Control | Patient | Control |
| Gleason score |  |  |  |  |  |  |
| <7 | 42.0% | - | 42.5% | - | 45.5% | - |
| 7 | 47.0% | - | 46.5% | - | 46.0% | - |
| >7 | 11.0% | - | 11.0% | - | 8.5% | - |
| Age  <55  55-59  60-64  65-69  ≥ 70 | 5.9%  12.1%  26.5%  33.5%  22.1% | 6.7%  11.3%  22.9%  32.4%  26.7% | 5.9%  13.0%  25.7%  33.1%  22.3% | 8.0%  10.3%  27.0%  29.2%  25.5% | 4.6%  12.3%  26.7%  32.4%  23.9% | 8.1%  9.7%  25.1%  28.6%  28.6% |
| BMI  <25 | 28.5% | 29.1% | 27.5% | 29.5% | 27.6% | 28.5% |
| 25-29 | 47.1% | 46.6% | 49.7% | 46.3% | 51.3% | 52.0% |
| ≥30 | 22.4% | 24.3% | 22.7% | 24.2% | 21.1% | 19.5% |

Supplementary table. Profile of subjects from the EPICAP cohort contacted and included in this study.
